# Transcrestal sinus lift and implant placement using the sinus balloon technique

**DOI:** 10.4317/medoral.17268

**Published:** 2011-12-06

**Authors:** María Peñarrocha-Diago, Sónnica Galán-Gil, Celia Carrillo-García, David Peñarrocha-Diago, Miguel Peñarrocha-Diago

**Affiliations:** 1 Associate Professor of Oral Surgery. Valencia University Medical and Dental School. Valencia; 2 Master of Oral Surgery and Implantology. Valencia University Medical and Dental School. Valencia; 3 Student Master of Oral Surgery and Implantology. Valencia University Medical and Dental School. Valencia; 4 Chairman of Oral Surgery. Director of the Master of Oral Surgery and Implantology. Valencia University Medical and Dental School. Valencia (Spain)

## Abstract

Objective: A description is made of transcrestal sinus lift using the sinus balloon technique, evaluating the bone height achieved and implant success one year after prosthetic loading. Material and method: Between January and July 2007, transcrestal sinus lift using the sinus balloon technique for dental implant placement was carried out in 6 patients. A panoramic X-ray study and maxillary computed tomography scan were carried out before the operation, in order to discard possible sinus pathology. During the intervention, the integrity of the sinus membrane was evaluated using a Medi Pack Pal endoscope (Farol Store and Co., Tuttlingen, Germany), and the intraoperative complications were analyzed. The dental implants were placed in the same surgical step in the presence of 3 mm or more of residual bone. Following the operation, panoramic X-rays were used to assess the bone height gained. One year after prosthetic loading, the implant success rate was determined based on the criteria of Buser. Results: One patient was excluded due to Schneider’s membrane perforation as confirmed by endoscopy. Trans-crestal sinus lift was carried out in 5 males with a mean age of 41.6 years (range 27-51), without antecedents of sinus disease. There were no intraoperative complications. In four patients the implants were placed simultaneous to sinus lift, while in another case implant placement was postponed due to insufficient remaining bone height. The mean gain in height after the operation was 8.7 mm. One year after prosthetic loading, the implant success rate was 100%. Conclusions: Transcrestal sinus lift using the sinus balloon technique is a minimally invasive procedure. In 5 patients the bone height gained proved sufficient to allow implant placement even in the presence of 3 mm of residual bone.

** Key words:** Sinus lift, balloon, sinus complications.

## Introduction

Transcrestal sinus lift using the sinus balloon technique is based on the osteotome transcrestal procedure described by Summers in 1994. The advantage of the balloon technique is that it can be used in the presence of 3 mm or more of residual bone (1), while conventional transcrestal lift using osteotomes requires a minimum of 6 mm of residual crestal bone (2).

Muronoi et al. in 2003 (3), and Soltan et al. in 2005 (4) described the use of the sinus balloon in direct sinus lift, placing it through a window in the lateral sinus wall. Kfir et al. in 2006 (5) described transcrestal sinus lift using the sinus balloon technique, placing bone grafts and dental implants in the same surgical step. Since then a number of articles have been published, with the contribution of 112 patients in 2009 (1,5-7). Other authors such as Hu et al. (8) have also used this technique to place implants in the same step as transcrestal sinus lift, with excellent results .

The present study describes transcrestal sinus lift using the sinus balloon technique for implant placement in patients with upper maxillary posterior sector atrophy, evaluating the bone height achieved and the implant success rate one year after prosthetic loading. 

## Material and Methods

 -Patients 

Between January and July 2007, transcrestal sinus lift using the sinus balloon technique for dental implant placement was carried out in 6 patients. 

We included patients with edentulous gaps in the antral zone of the upper maxillary posterior sector, with sufficient bone height to place implants using the conventional protocol (< 8 mm) and/or indirect lift using osteotomes (<6 mm). The absence of maxillary sinus disease antecedents was confirmed by computed tomography (CT) and panoramic X-rays, and the minimum follow-up period after treatment was one year. Patients showing membrane perforation during the operation were excluded. 

The patient clinical data were compiled based on a previously established protocol: age, gender, disease antecedents, smoking, characteristics of the edentulous space, and duration of follow-up.

 -Surgical protocol

Surgery was performed under local anesthesia (4% articaine with 1:100,000 adrenalin as vasoconstrictor). A full thickness flap was raised and the bed was prepared using drills and osteotomes worked to within 1 mm from the floor of the maxillary sinus. An osteotome tip was then inserted, and gentle tapping was applied to allow controlled fracture of the sinus cortical layer. 

Posteriorly, the integrity of the Schneider’s membrane was evaluated using a Medi Pack Pal endoscope (Farol Store and Co., Tuttlingen, Germany) inserted through the implant bed.

The latex balloon was fitted to a catheter used to insufflate the balloon. Before placing it within the bone bed, correct functioning of the balloon was checked by insufflating it several times. The balloon was inserted in the subantral space, performing progressive, slow and controlled insufflations with saline solution. This procedure was repeated several times, taking care never to introduce more than 4 ml each time. During the insufflation sequence we used the endoscope to check the condition of the membrane. 

The sinus membrane was detached to the desired height, and in all cases we introduced Biooss® particulate, freeze-dried bovine bone grafts (Geistlich Pharma AG. Switzerland) together with the autologous bone shavings obtained from drilling. 

The dental implants were placed in the same surgical step in the presence of 3 mm or more of residual maxillary alveolar crestal bone. In those cases where the implants were not placed in the same surgical step, we waited three months to allow bone graft consolidation before implant placement. After surgery, all patients received amoxicillin-clavulanic acid (Clamoxyl®, GlaxoS-mithKline, Madrid, Spain) 875/125 mg every 8 hours for 7 days, ibuprofen (Bexistar®, Laboratorio Bacino, Barcelona, Spain) 600 mg every 8 hours for 4 days, and 0.12% chlorhexidine rinses (GUM, John O. Butler/Sunstar, Chicago, IL, USA) three times a day for 7 days.

Second step surgery was performed three months after implant placement, and prosthesis placement in turn was carried out one month after second step surgery.

 -Measurement of bone gain

**Figure 1:**
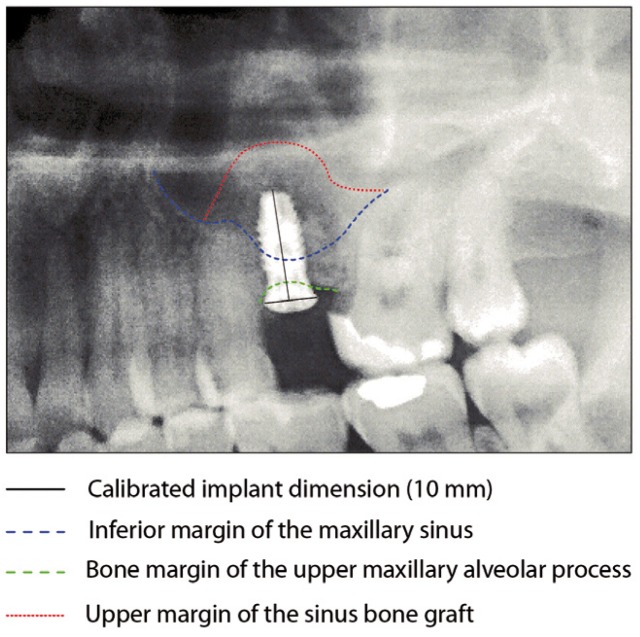
Measurement of bone height gained from the postoperative panoramic X-rays. Calibration of the X-rays based on the placed implant. panoramic X-rays. Calibration of the X-rays based on the placed implant.

**Figure 2:**
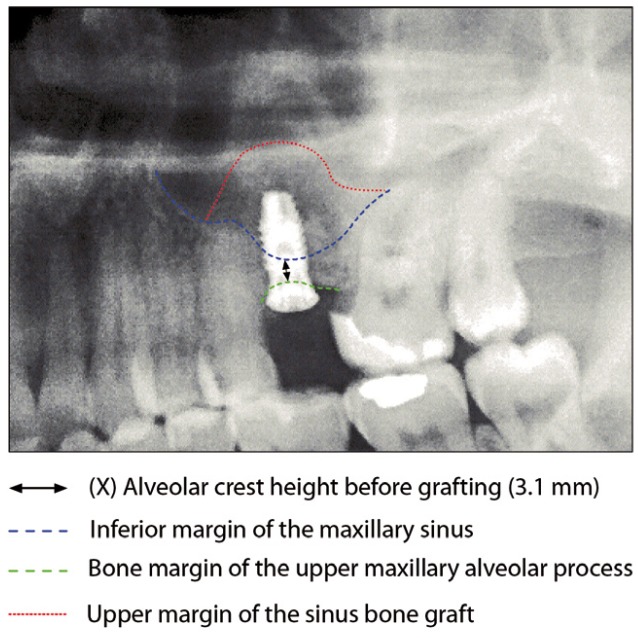
Measurement of bone height gained from the postoperative panoramic X-rays. Bone height before the operation. panoramic X-rays. Bone height before the operation.

**Figure 3:**
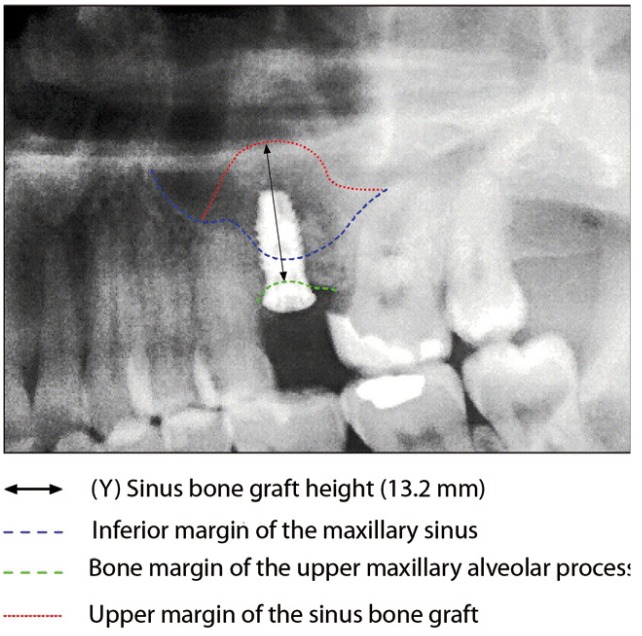
Measurement of bone height gained from the postoperative panoramic X-rays. Total maxillary sinus elevation.

Following the operation, panoramic X-rays were used to assess the bone height gained with sinus lift. The bone margin of the upper maxillary alveolar process was traced, together with the lower margin of the maxillary sinus and the upper margin of the bone graft (Fig. 1). Panoramic X-ray distortion was calculated based on a known measure: the length of the positioned implants (Fig. 1). After X-ray calibration, a vertical line was traced at the implant axis from the bone margin of the upper maxillary alveolar process to the floor of the maxillary sinus, to calculate the bone height prior to graft placement (measure X)(Fig. 2). Likewise, we measured the distance from the margin of the alveolar process to the upper margin of the positioned bone graft, thus calculat-ing the total sinus lift height (measure Y)(Fig. 3). The difference between the two measures (Y-X) represented the bone height gained.

The Student t-test for related samples was used to correlate the mean initial residual crest height to the bone height gained by sinus lift one year after prosthesis placement.

 -Percentage success

One year after prosthetic loading, the implant success rate was determined based on the criteria of Buser et al. 1999 (9).

## Results

One patient was excluded due to Schneider’s membrane perforation as confirmed by endoscopy. Transcrestal sinus lift was carried out in 5 males with a mean age of 41.6 years (range 27-51), without antecedents of sinus disease. There were no intraoperative complications.

 Table 1 shows the clinical data of the 5 treated subjects (age, gender, sinus disease antecedents, smoking and characteristics of the edentulous space), the implants and intervention.

In cases 1, 2, 3 and 4 the implants were placed at the same time as sinus lift (Figs. 4, 5, 6, 7, 8, 9, 10, 11, 12). In case 5 the remaining bone height (2.1 mm) proved insufficient for implant placement. Sinus lift and bone grafting were carried out, and we postponed implant placement for three months (Figs. 13, 14, 15, 16, 17, 18, 19, 20).

The mean gain in bone height after the operation was 8.7 mm. The general data relating to gained height are reported in  Table 2. The results obtained with the Student t-test for related samples revealed a statistically significant gain in bone height (increase = 9.32 mm; p<0.001) – the mean initial height being 3.38 mm (SD = 0.81), while the mean height one year after the operation was 12.70 mm (SD = 1.25) (Fig. 21).

One year after prosthetic loading, none of the patients showed symptoms of sinus disease. According to the criteria of Buser et al., the implant success rate was 100% in the 5 patients.

**Figure 4:**
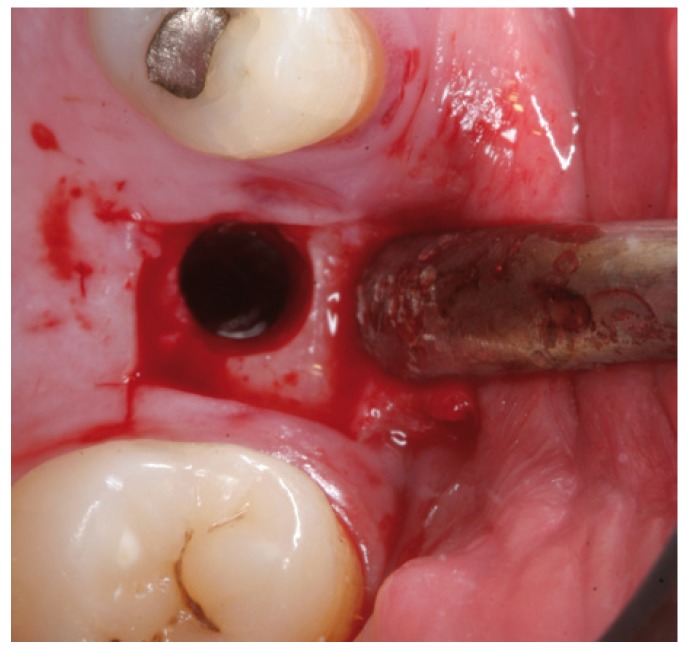
Case 1. 1. Full thickness flap elevation and preparation of the implant bed with drills and osteotomes.

**Figure 5:**
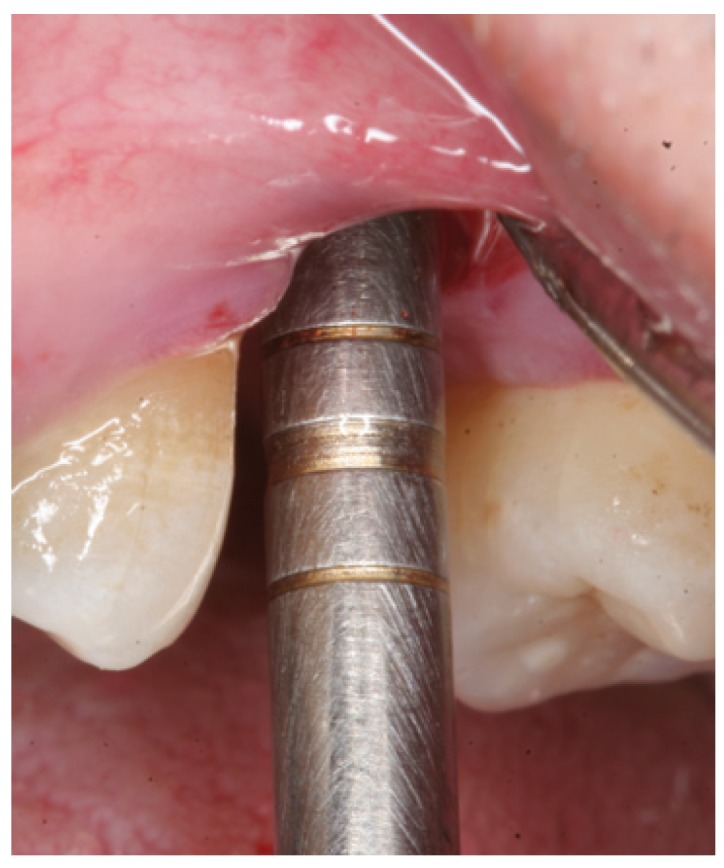
Case 1. 2. Controlled fracture of the floor of the maxillary sinus using an osteotome.

**Figure 6:**
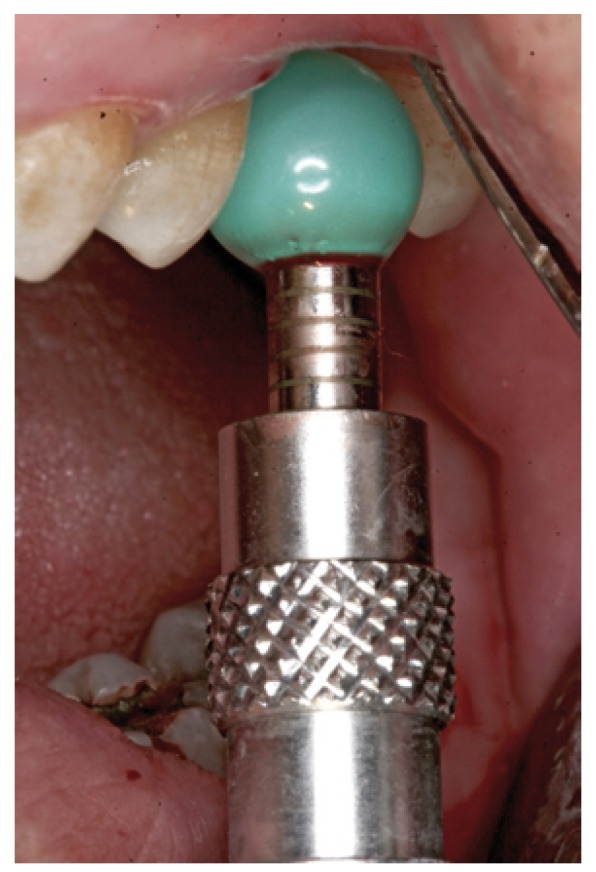
Case 1. 3. Insertion of the sinus balloon for detachment of the sinus membrane.

**Figure 7:**
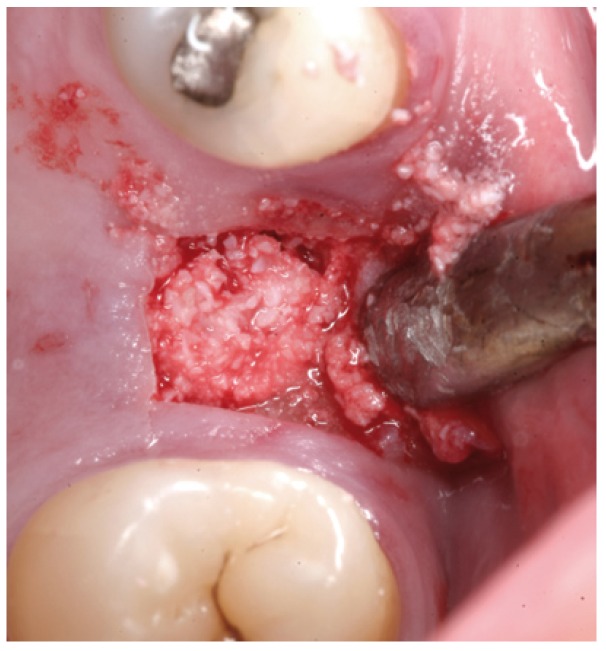
Case 1. 4. Particulate bone graft placement.

**Figure 8:**
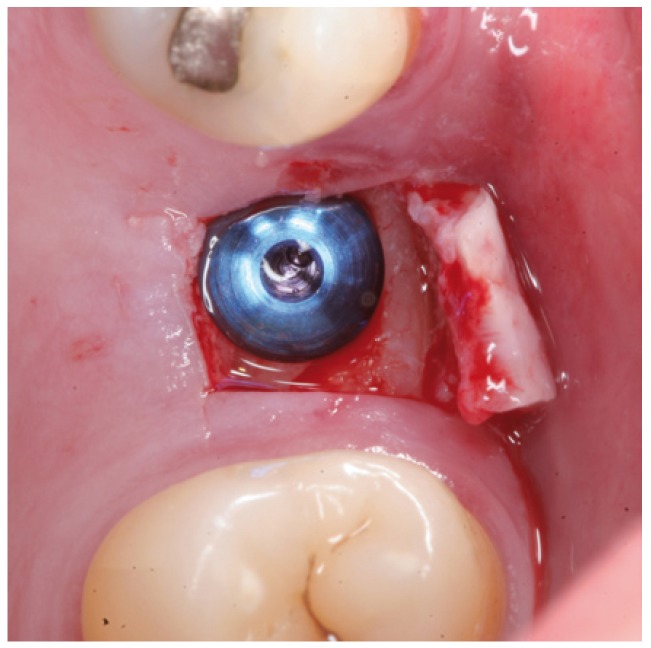
Case 1. 5. Implant positioned in the same surgical step.

**Figure 9:**
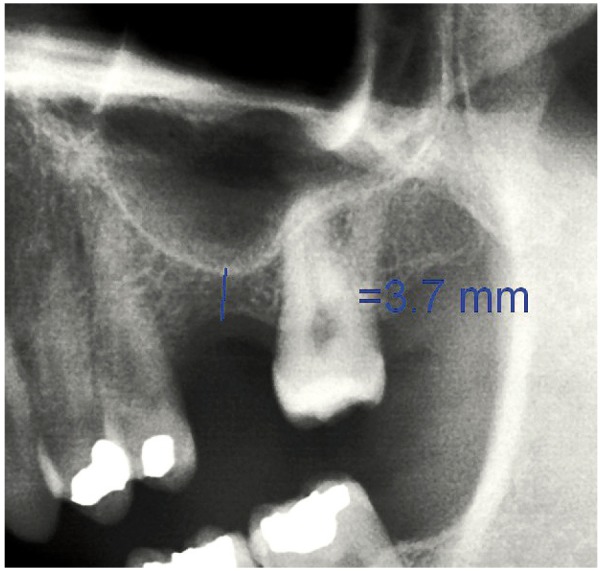
Case 1. 6. Preoperative panoramic X-ray view.

**Figure 10:**
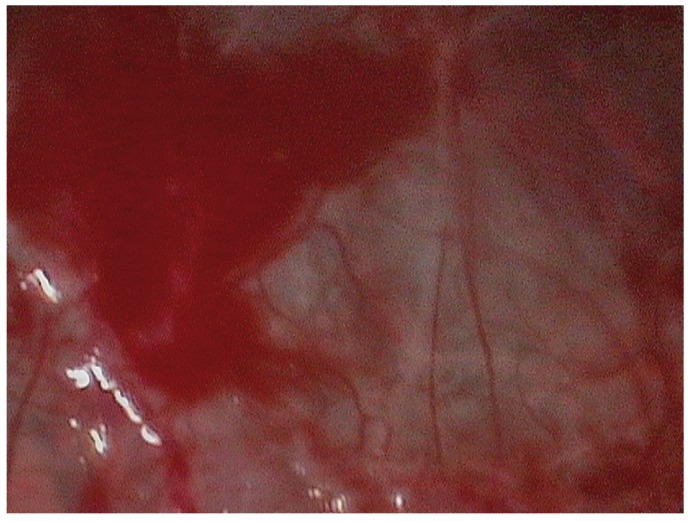
Case 1. 7. Sinus membrane integrity as established by endoscopy.

**Figure 11:**
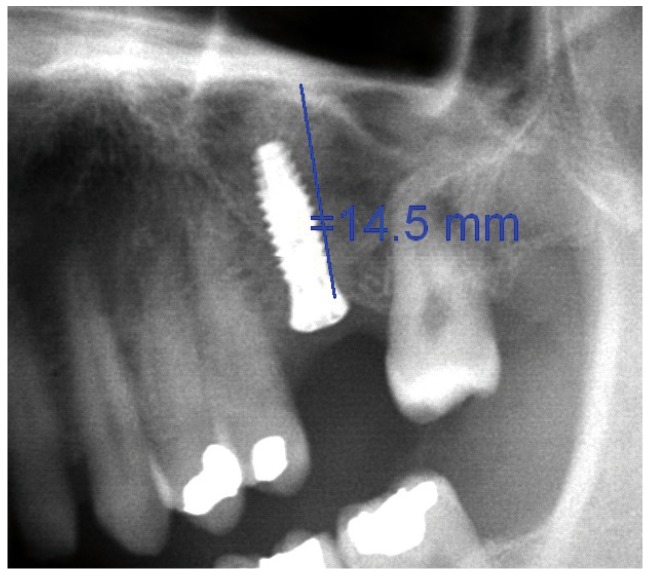
Case 1. 8. Postoperative panoramic X-ray view.

**Figure 12:**
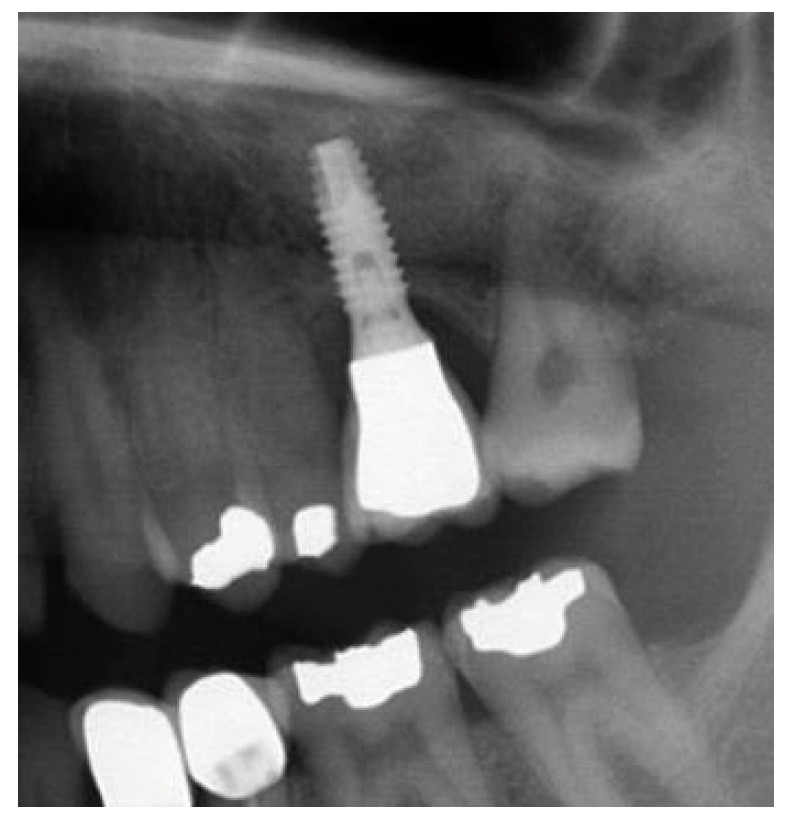
Case 1. 9. Panoramic X-ray view one year after prosthetic loading.

**Figure 13:**
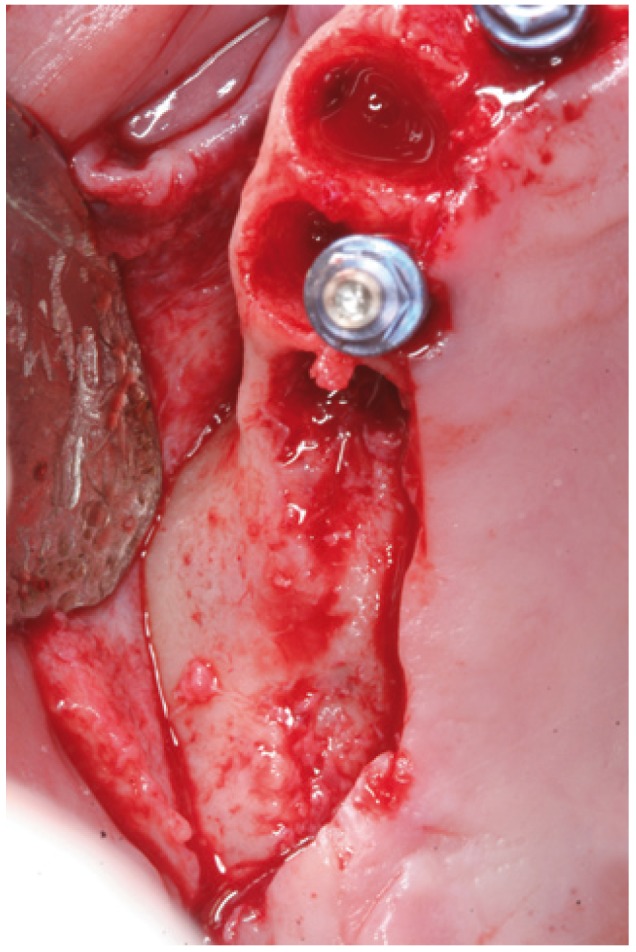
Case 2. 1. Full thickness mucoperiosteal flap for gaining access to the maxillary alveolar crest.

**Figure 14:**
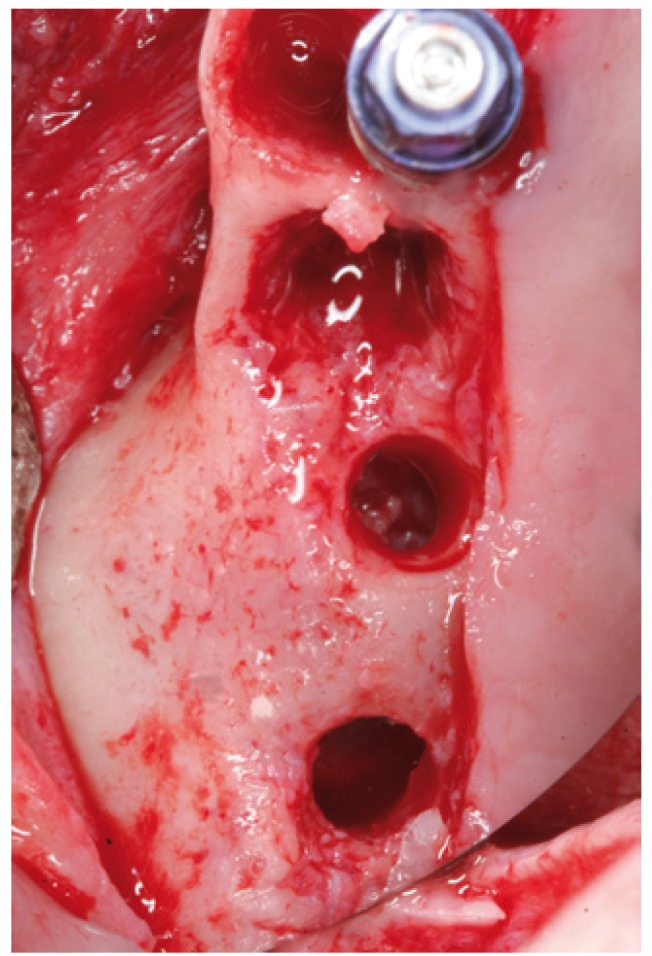
Case 2. 2. Preparation of the implant bed with drills and osteotomes.

**Figure 15:**
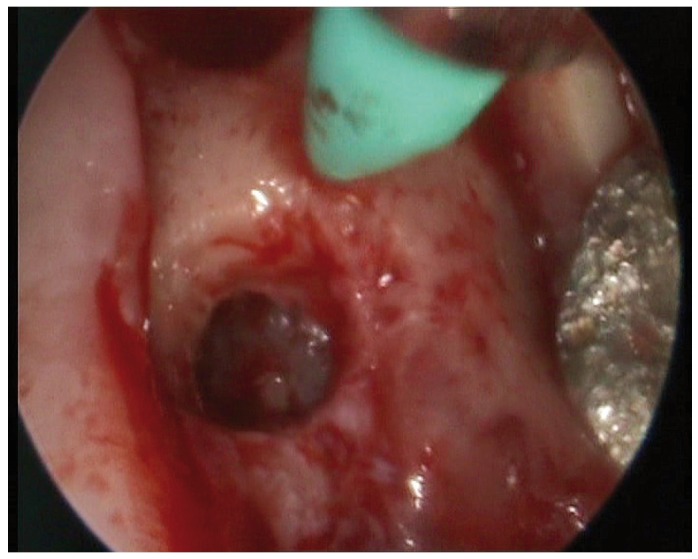
Case 2. 3. Insertion of the latex balloon and checking of sinus membrane condition.

**Figure 16:**
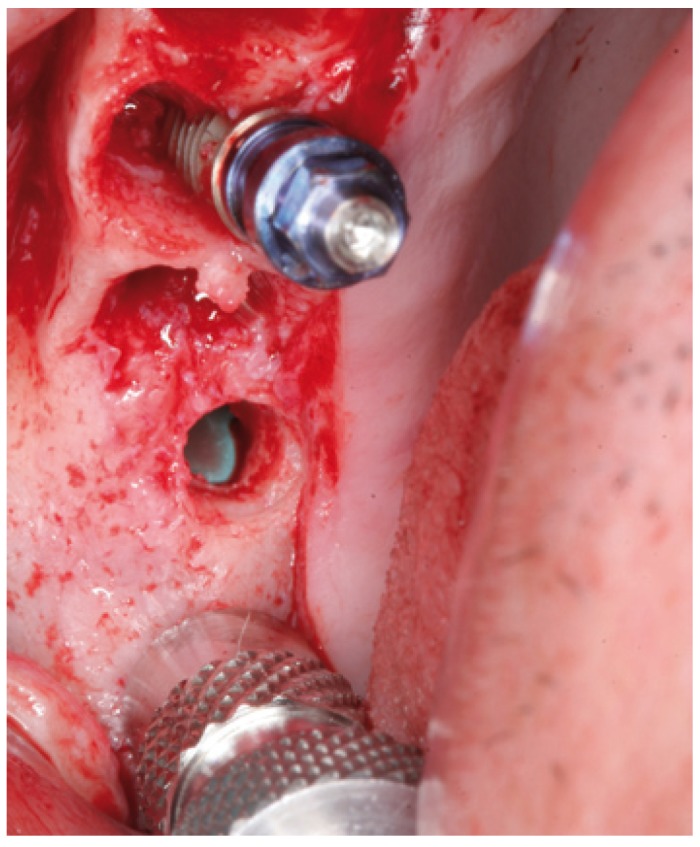
Case 2. 4. Sinus balloon within the maxillary sinus.

**Figure 17:**
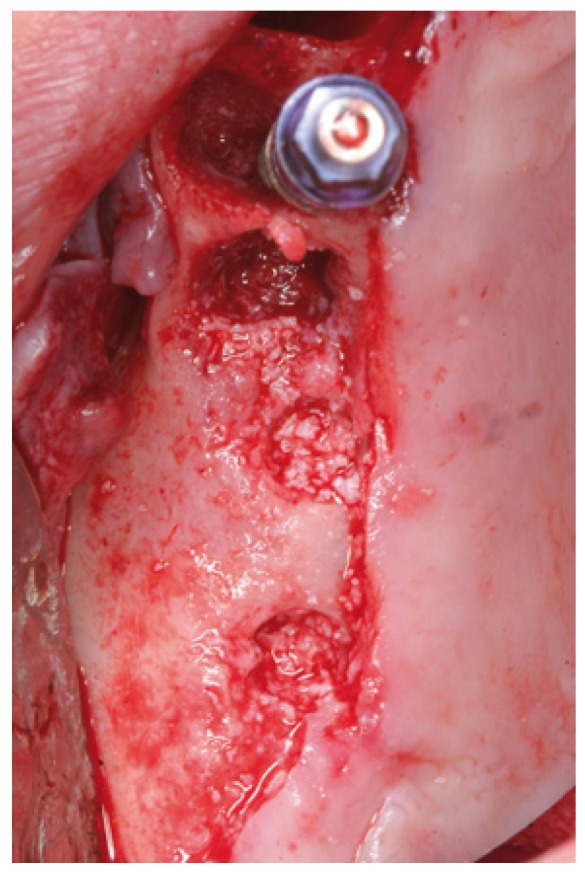
Case 2. 5. Particulate bone graft placement.

**Figure 18:**
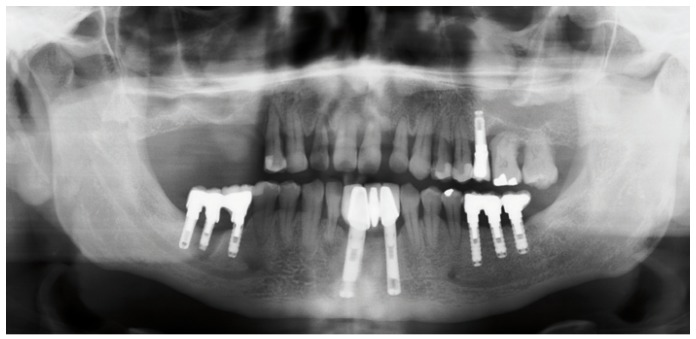
Case 2. 6. Preoperative panoramic X-ray view.

**Figure 19:**
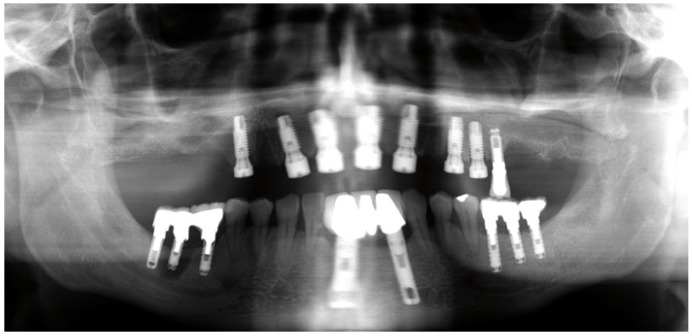
Case 2. 7. Postoperative panoramic X-ray view after sinus lift with the balloon technique. Implant placement at this level was postponed three months due to an insufficient initial alveolar crest height (2.1 mm).

**Figure 20:**
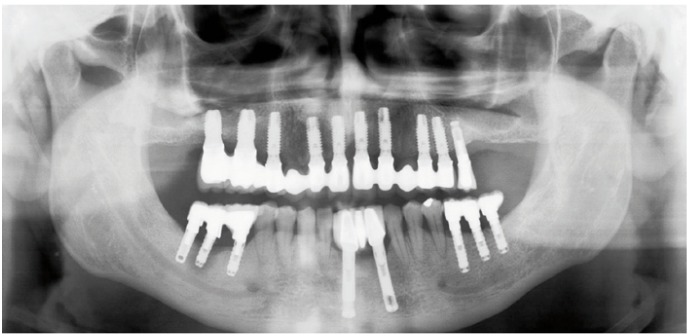
Case 2. 8. Panoramic X-ray view with the implants and prosthesis, one year after loading.

**Figure 21:**
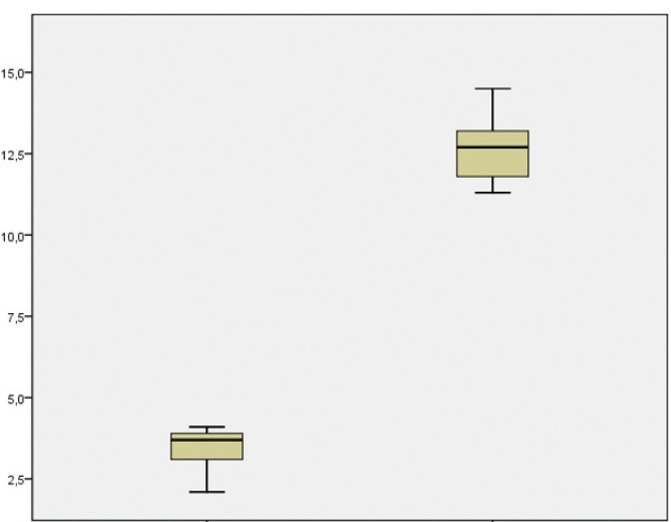
Statistical results relating to bone height gained one year after prosthetic loading.

**Table 1:**
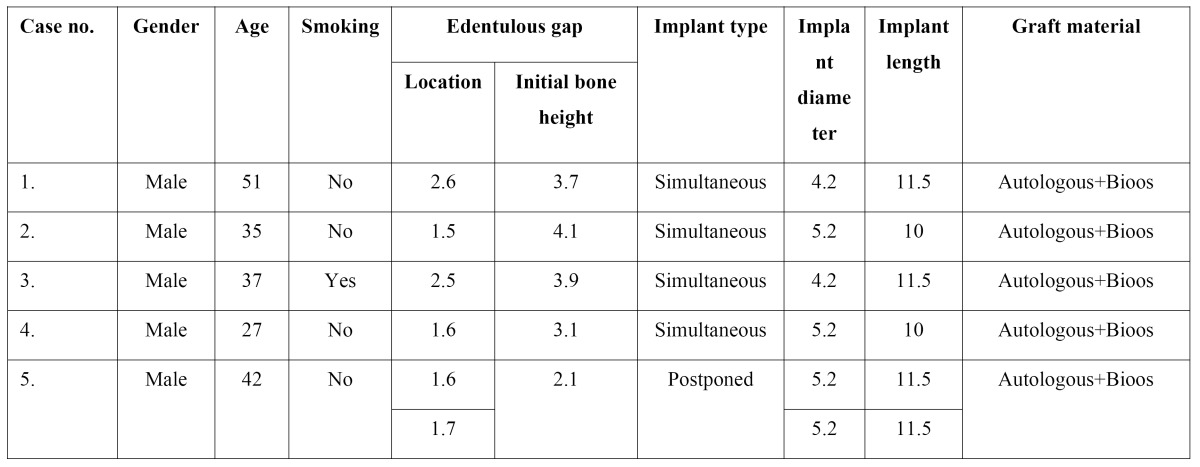
Patient and implant characteristics.

**Table 2:**
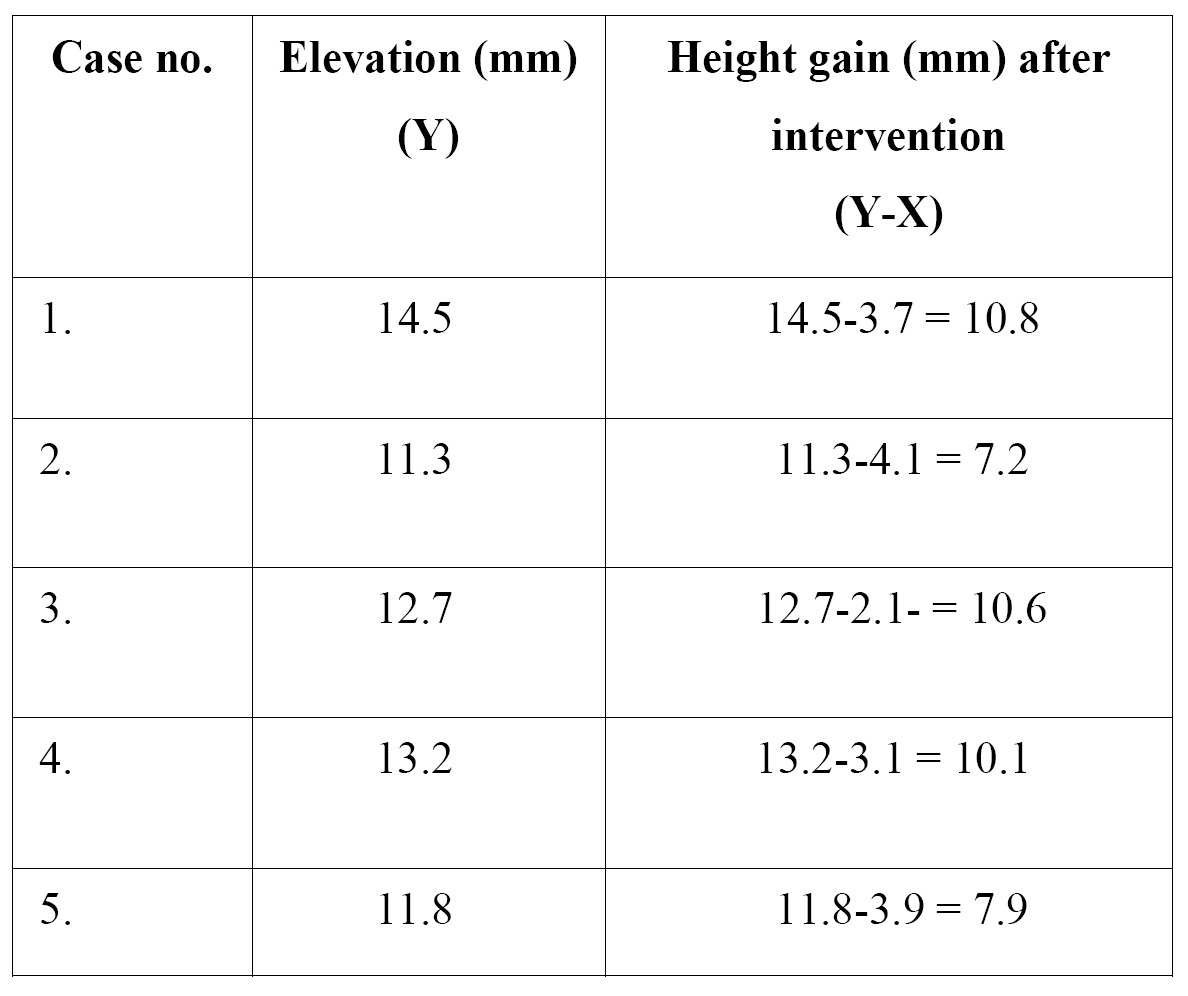
Characteristics of the height gained.

## Discussion

Transcrestal sinus lift using the sinus balloon technique involves the insertion of a latex balloon through the alveolar crest, insufflating it with saline solution through a catheter in order to detach the sinus membrane. 

The perforation of this membrane is one of the most frequent intraoperative complication of such sinus lift procedures (2). In the present study, endoscopy revealed sinus membrane rupture in one of the patients, leading to discontinuation of the procedure. However, authors such as Hu et al. (8) and Stelzle et al. (10) consider the complications rate with this technique to be low. In 2009, Stelzle et al. (10) evaluated four indirect sinus lift methods designed to afford crest increments of up to 10 mm (sinus lift with osteotomes, sinus lift with bone grafts and osteotomes, sinus lift with ultrasound, and sinus lift with the sinus balloon). The sinus balloon technique was found to be the best option for sinus lifting up to 10 mm, and moreover produced the fewest intraoperative complications.

The advantage of the balloon technique is that it can be applied to alveolar crests measuring 3 mm or less (1) – in contrast to the classical indirect technique with osteotomes, where the minimum acceptable crest height is 6 mm (2). This is because sinus lift with osteotomes affords a height gain of 3 ± 0.8 mm (11), while the balloon technique can afford sinus membrane elevations of up to 10 mm (10). In the present study we used the sinus balloon technique to treat 5 patients with a remaining bone height of 3.8 mm (range 2.1-4.1 mm). Similar results were obtained by Kfir et al. (7), in a multicenter study of 112 patients subjected to transcrestal sinus lift using the sinus balloon technique, in which the mean remaining crest height was 3.8 ± 2.1 mm. The mean gain in height after the operation was 8.7 mm, allowing implant placement in the same surgical step.

## Conclusion

Transcrestal sinus lift using the sinus balloon technique is a minimally invasive procedure involving few intraoperative complications. In the present study we were able to perform transcrestal sinus lift from 3 mm of residual bone, gaining a mean height of up to 8.7 mm, and with a 100% implant success rate one year after prosthetic loading.

## References

[1] Kfir E, Goldstein M, Rafaelov R, Yerushalmi I, Kfir V, Mazor Z (2009). Minimally invasive antral membrane balloon elevation in the presence of antral septa: a report of 26 procedures. J Oral Implantol.

[2] Emmerich D, Att W, Stappert C (2005). Sinus floor elevation using osteotomes: a systematic review and meta-analysis. J Periodontol.

[3] Muronoi M, Xu H, Shimizu Y, Ooya K (2003). Simplified procedure for augmentation of the sinus floor using a haemostatic nasal balloon. Br J Oral Maxillofac Surg.

[4] Soltan M, Smiler DG (2005). Antral membrane balloon elevation. J Oral Implantol.

[5] Kfir E, Kfir V, Mijiritsky E, Rafaeloff R, Kaluski E (2006). Minimally invasive antral membrane balloon elevation followed by maxillary bone augmentation and implant fixation. J Oral Implantol.

[6] Kfir E, Kfir V, Eliav E, Kaluski E (2007). Minimally invasive antral membrane balloon elevation: report of 36 procedures. J Periodontol.

[7] Kfir E, Goldstein M, Yerushalmi I, Rafaelov R, Mazor Z, Kfir V (2009). Minimally invasive antral membrane balloon elevation - results of a multicenter registry. Clin Implant Dent Relat Res.

[8] Hu X, Lin Y, Metzmacher AR, Zhang Y (2009). Sinus membrane lift using a water balloon followed by bone grafting and implant placement: a 28-case report. Int J Prosthodont.

[9] Buser D, Weber HP, Lang NP (1990). Tissue integration of non-submerged implants 1-year results of a prospective study with 100 ITI hollow-cylinder and hollow-screw implants. Clin Oral Implants Res.

[10] Stelzle F, Benner KU (2011). Evaluation of different methods of indirect sinus floor elevation for elevation heights of 10mm: an experimental ex vivo study. Clin Implant Dent Relat Res.

[11] Nkenke E, Schlegel A, Schultze-Mosgau S, Neukam FW, Wiltfang J (2002). The endoscopically controlled osteotome sinus floor elevation: a preliminary prospective study. Int J Oral Maxillofac Implants.

